# Interaction of ARF-1.1 and neuronal calcium sensor-1 in the control of the temperature-dependency of locomotion in *Caenorhabditis elegans*

**DOI:** 10.1038/srep30023

**Published:** 2016-07-20

**Authors:** Paul A. C. Todd, Hannah V. McCue, Lee P. Haynes, Jeff W. Barclay, Robert D. Burgoyne

**Affiliations:** 1 Department of Cellular and Molecular Physiology, The Physiological Laboratory, Institute of Translational Medicine, University of Liverpool, Crown Street, Liverpool, L69 3BX, United Kingdom

## Abstract

Neuronal calcium sensor-1 (NCS-1) mediates changes in cellular function by regulating various target proteins. Many potential targets have been identified but the physiological significance of only a few has been established. Upon temperature elevation, *Caenorhabditis elegans* exhibits reversible paralysis. In the absence of NCS-1, worms show delayed onset and a shorter duration of paralysis. This phenotype can be rescued by re-expression of *ncs-1* in AIY neurons. Mutants with defects in four potential NCS-1 targets (*arf-1.1*, *pifk-1*, *trp-1* and *trp-2*) showed qualitatively similar phenotypes to *ncs-1* null worms, although the effect of *pifk-1* mutation on time to paralysis was considerably delayed. Inhibition of *pifk-1* also resulted in a locomotion phenotype. Analysis of double mutants showed no additive effects between mutations in *ncs*-1 and *trp-1* or *trp-2*. In contrast, double mutants of *arf-1.1* and *ncs-1* had an intermediate phenotype, consistent with NCS-1 and ARF-1.1 acting in the same pathway. Over-expression of *arf-1.1* in the AIY neurons was sufficient to rescue partially the phenotype of both the *arf-1.1* and the *ncs-1* null worms. These findings suggest that ARF-1.1 interacts with NCS-1 in AIY neurons and potentially *pifk-1* in the Ca^2+^ signaling pathway that leads to inhibited locomotion at an elevated temperature.

Many aspects of neuronal function and subsequent changes in organism behaviour are regulated by changes in the concentration of intracellular free calcium ([Ca^2+^]_i_) in the neurons. Changes in [Ca^2+^]_i_ are transduced through Ca^2+^ sensor proteins and neurons express a large number of these proteins^1^ including synaptotagmin I which is required for fast neurotransmission^2^, and many different EF-hand containing proteins^1^ related to the ubiquitous Ca^2+^ sensor protein calmodulin^3^. Calmodulin has many functions in neurons acting via targets such as Ca^2+^/calmodulin-dependent protein kinase II^4^. Other EF-hand containing Ca^2+^ sensors expressed in neurons include the calcium-binding protein (CaBP) family^5^,^6^ and the neuronal calcium sensor (NCS) protein family^7^,^8^,^9^.

Ca^2+^ sensor proteins undergo a conformational change on Ca^2+^ binding that results in a change in their interaction and regulation of one or more target effector proteins. The specificity of neuronal Ca^2+^ signaling is in large part determined by differential signal processing by these Ca^2+^ sensor proteins through their specific target proteins^3^,^10^,^11^. The rules underlying the sensing and specificity of Ca^2+^ signaling in neurons have begun to emerge in recent years but still remain to be fully understood.

The Neuronal Calcium Sensor (NCS) family of proteins consists of 5 sub-families including Class A (NCS-1/frequenin), Class B (visinin-like proteins (VILIPs), Class C (recoverin), Class D (guanylate cyclase activating proteins (GCAPs) and Class E (K^+^ channel interacting proteins (KChIPs)^7^. NCS-1 was first identified in *Drosophila*^12^ but orthologues of NCS-1 have been subsequently identified in all eukaryotes, from yeast to man. Genetic and functional studies have determined that NCS-1 has multiple physiological functions^8^,^13^,^14^,^15^. In *Saccharomyces cerevisiae*, NCS-1 (Frq1) is essential for survival due to its requirement for activation of the phosphatidylinositol-4-kinase enzyme Pik1^16^ although its absence is not lethal in other organisms. Some of the roles of NCS-1 may also be specific to particular organisms such as its specific role in temperature-dependent behaviours in *Caenorhabditis elegans* as a consequence of its restricted neuronal expression^17^,^18^,^19^. In mammalian cells NCS-1 regulates Ca^2+^ -dependent exocytosis^20^, long-term depression^21^ axonal growth and neuronal regeneration^22^ and channel function^23^,^24^. Studies at an organism level have identified key NCS-1 functions^8^,^9^. In mice, knock-out of NCS-1 has relatively subtle effects but results in an increase in anxiety and depressive behaviour^25^ and over-expression of NCS-1 in adult mouse dentate gyrus neurons affected exploratory behaviour and the acquisition of spatial memory by regulating the surface expression of dopamine D2 receptors in the hippocampus^26^. In *C. elegans* NCS-1 is expressed in sensory neurons and interneurons and is involved in neuronal pathways that control long term memory for thermosensation^17^ and has also been implicated in chemotaxis^27^.

NCS-1 has many identified interacting partners^28^ some of which are unique for NCS-1 but others that are also regulated by other Ca^2+^ sensors particularly calmodulin^29^. There are some physiological effects of NCS-1 that can be attributed directly to one of its identified target proteins. The NCS-1 target proteins linked to function include phosphatidylinositol-4-kinase (PI4K) IIIβ^30^,^31^ and its orthologue Pik1 in yeast^16^, interleukin receptor accessory protein like-1 (IL1RAPL1)^32^, TRPC5 channels^33^, InsP(3) receptors^34^, Ric8a^35^, P/Q-type Ca^2+^ channels^23^,^36^,^37^ and dopamine D2 and D3 receptors^38^,^39^,^40^. Some of the protein interactions are known, however, only from *in vitro* binding assays and so their biological importance is unclear. It is possible, for example, that those binding partners that are also calmodulin targets *in vitro* are not regulated by NCS-1 under physiological conditions^41^.

One mammalian NCS-1 binding partner is ARF1^30^,^42^, which was found to bind to NCS-1 in a Ca^2+^ -dependent manner, and interestingly both ARF1 and NCS-1 directly interact with and stimulate the activity of PI4KIIIβ. In non-neuronal cells over-expression of ARF1 reversed the inhibitory effect of NCS-1 on traffic from the Golgi complex, suggesting the possibility of a functionally relevant interaction between the two proteins^30^. Subsequently, both NCS-1 and ARF-1 were found to be required for inner ear development in zebrafish potentially via a common pathway^43^. To explore further whether an ARF1/NCS-1 interaction was required in a physiological context for an NCS-1-dependent effect, we took advantage of an assay for temperature-dependent changes in locomotion in *C. elegans*^44^, in which NCS-1 is required to be expressed in the pair of AIY neurons^19^. Analysis of worms with mutations in various potential NCS-1 target proteins established a potential role for *pifk-1*, the orthologue of PI4KIIIβ, *trp-1*, *trp*-2 and *arf-1.1* in the temperature-dependent locomotion (TDL) assay. In particular, we observed a genetic interaction between *arf-1.1* and *ncs-1*. ARF1.1 function in the TDL assay required its expression in AIY neurons. These findings suggest that both ARF1.1 and NCS-1 are components of the same temperature-dependent Ca^2+^ signaling pathway in AIY neurons.

## Results

### Characterisation of a modified temperature-dependent locomotion assay

**Figure 1:**
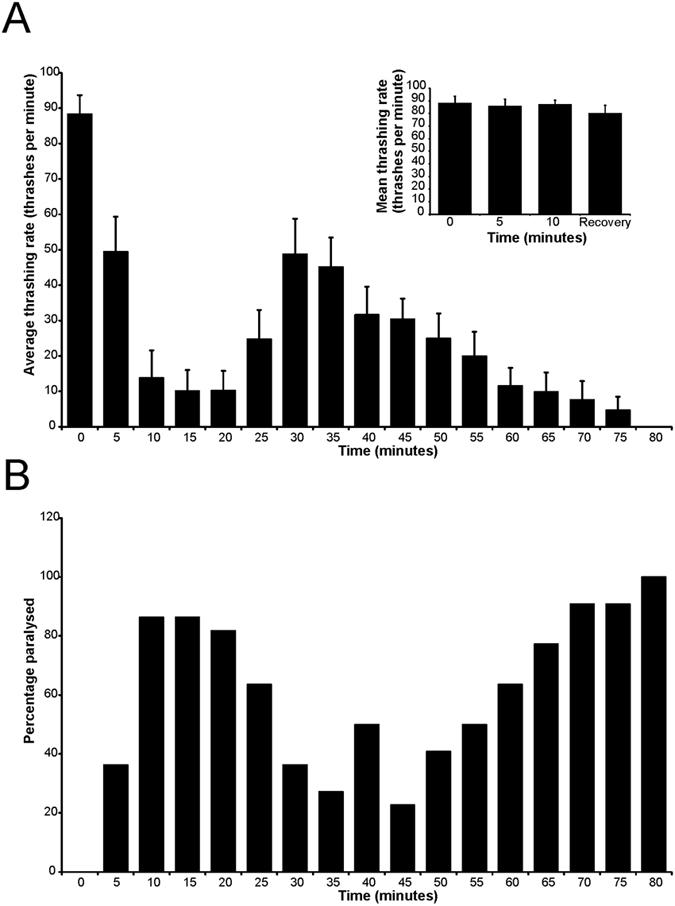
Changes in locomotion over time following temperature elevation. N2 worms were examined following elevation of temperature from 20 °C ± 0.5 to a final temperature of 28.0 °C ± 0.5. The rate of thrashing of individual worms was determined and the data shown as the mean rate of thrashing for the population ± SEM (**A**). The inset shows the rate of thrashing of those worms that were not paralysed, showing that they thrashed at the same rate before and during the time when other worms became paralysed and after the initial recovery period. The percentage of worm that were paralysed at each time point was also determined (**B**).

Our previous work showed that acute elevation of temperature from 20 °C to 28 °C for 10 mins resulted in a significant slowing of movement of wild-type (N2) worms^44^. In contrast, *ncs-1* null worms (*qa401*) showed not a slowing but instead a small but significantly faster rate of movement at the elevated temperature^19^. NCS-1 is normally expressed in the pair of AIY neurons^17^, which are involved in the known circuit that functions in thermotaxis in the worm^45^. The TDL phenotype in the *ncs-1* null worms was completely reversed by the pan-neuronal expression of *ncs-1*, driven by a *Prab-3* promoter, or the AIY-specific expression of *ncs-1*, driven by a *Pttx*-3 promoter^19^. In order to develop a more detailed and potentially more informative assay, we examined worm thrashing at an elevated temperature in real-time. We determined that in response to elevated temperature, worm thrashing rate decreased; however, this was a reflection of a proportion of worms undergoing temperature-dependent paralysis (Fig. 1A,B). In the assay of thrashing in N2 worms, it was observed that more than 80% of worms were paralysed within 10 mins of temperature elevation (Fig. 1A,B). Surprisingly, however, many of the worms showed a temporary recovery to normal thrashing behaviour before becoming paralysed again at later times (Supplementary video 1). It was observed at various times during the assay that those worms that were not paralysed moved at a similar rate, indicating that the paralysis was an all-or-none effect (Fig. 1A, inset). Based on these observations, we subsequently used a revised assay in which we determined the mean time to first paralysis and the duration of the first period of paralysis for individual worms within populations.

From initial experiments, an elevated temperature of 28.5 ± 0.5 °C was selected as optimal in showing consistency in worm responses. A temperature shift to 26.5 ^o^C resulted in an observable difference in time to paralysis of *ncs-1* null mutants versus N2 worms; but this represented paralysis of only 33% of the *ncs-1* null worms. In contrast, a shift to 30 °C showed little difference in time to paralysis between *ncs-1* null mutants and N2 worms (Supplementary Fig. 1).

**Figure 2:**
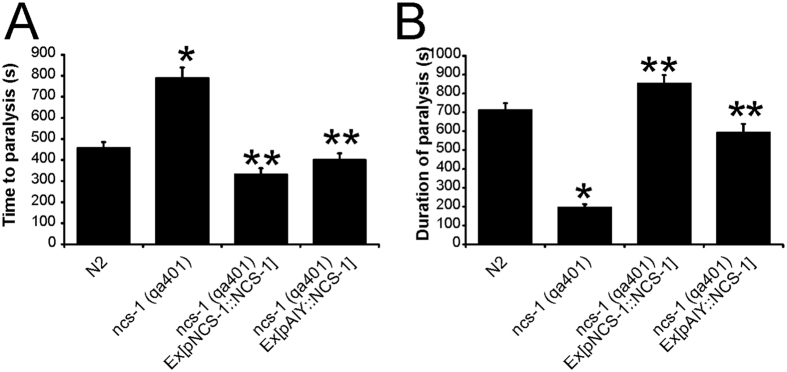
Analysis of the temperature-dependent locomotion of null *ncs-1* and *NCS-1* rescue strains. Various *C. elegans* strains, as indicated, were analysed using the TDL assay as described in the Methods section. Locomotion of worms was initially assessed at 20 °C and then following the temperature shift to 28.5 °C. Multiple animals were tested for each strain and mean values for time to paralysis (**A**) and the duration of paralysis (**B**) were determined and expressed as mean ± SEM. The numbers of animals used for each strain were N ≥ 30. All data sets were compared with N2 control worms and statistical differences for the onset and duration of paralysis for each strain was determined using one-way ANOVA and Dunnett’s correction for multiple comparisons (*p < 0.001 versus N2; **p < 0.001 versus *qa401*).

Use of the revised assay in which multiple individual worms were observed revealed that on average N2 worms became paralysed within 500s, but subsequently recovered after another 700s. The *ncs-1* null worms showed both an increase in the mean time to paralysis compared to N2 worms (73% increase) and a shorter (73% decrease) duration of paralysis (Fig. 2). Both aspects of this phenotype were rescued following expression of NCS-1 under its endogenous promoter. AIY-specific expression of NCS-1 also fully rescued the time to paralysis, while the duration of paralysis was only partially rescued with AIY-specific expression.

An important aspect for the subsequent analyses was that all worm strains assayed in this study showed similar basal thrashing rate before temperature elevation (Supplementary Table 1) and also all showed similar stereotypical thrashing behaviour.

### Assessment of the temperature-dependent locomotion of *C. elegans* strains with mutations in potential NCS-1 target proteins

As a first approach to identifying potential orthologues of Arf1 or other identified NCS-1 targets involved in this NCS-1-dependent pathway, we examined the phenotypes of various worm mutants in the revised TDL assay. We would argue that mutants of any downstream targets for NCS-1 should show a similar phenotype in the assay as *ncs-1* null worms, i.e., a longer time to paralysis and shorter duration of paralysis. A large number of potential NCS-1 interacting proteins are known particularly from the study of mammalian proteins^11^, some of which have orthologues in *C. elegans* (Supplementary Table 2). We excluded those candidates that do not have a clearly recognizable orthologue in *C. elegans* (e.g. IL1RAPL1). From the list of the existing worm orthologues, we then excluded any candidates that were known not to be expressed in AIY neurons (e.g., the dopamine D2 receptor orthologues *dop-2* and *dop-3*) or those where available mutants have very severe locomotion defects that would preclude their use in the TDL assay (e.g., the voltage-gated Ca^2+^ channel subunit *unc-2*). Following from this analysis we obtained mutant strains for the following target candidates: *pifk-1* (orthologue of mammalian PI4KIIIβ), *trp-1* and *trp-2* (orthologues of TRPC5), *arf-1.1* and *arf-1.2* (orthologues of ARF1) and *grk-2* (orthologue of GRK2).

**Figure 3:**
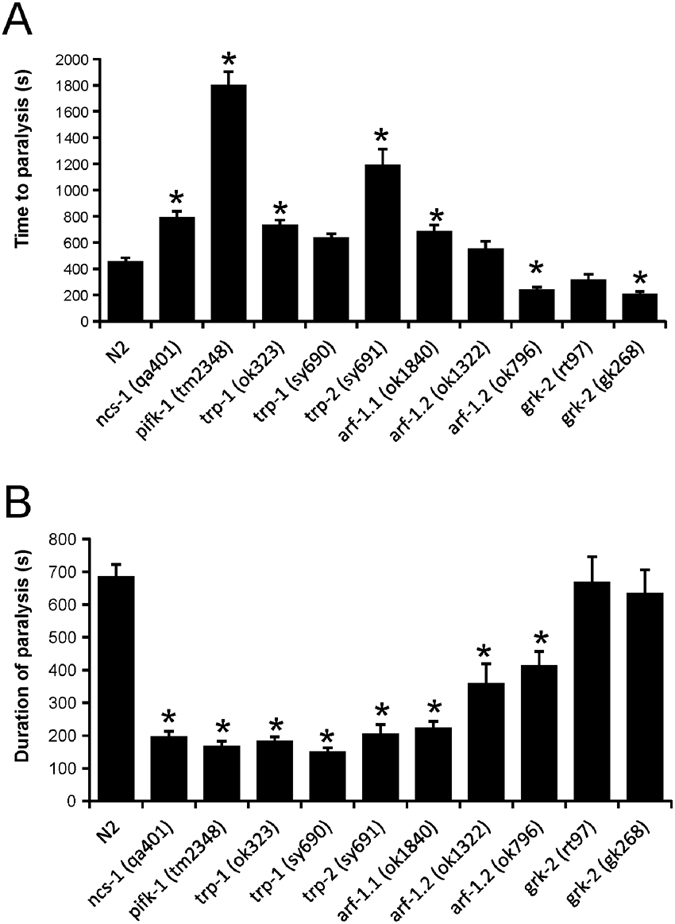
Assay of the temperature dependent locomotion of various mutant *C. elegans* strains. Temperature-dependent locomotion assays were carried out on various strains of *C. elegans*, as indicated, to provide mean values for the time to the start of paralysis after the shift to 28.5 °C and the duration of paralysis. Multiple animals were tested for each strain (N = ≥20) and mean values for time to paralysis (**A**) and the duration of paralysis (**B**) were determined. All data are expressed as mean ± S.E.M. Statistical differences were identified by comparing averaged data to that of N2 wild-type worms, using one-way ANOVA with Dunnett’s correction for multiple comparisons (*p < 0.05 versus N2).

Mutant strains were tested in the TDL assay in comparison with the behaviour of N2 and *ncs-1* null worms (Fig. 3). Mutant strains of *trp-1* (two separate alleles), *trp-2*, and *arf-1.1* showed changes in time of onset and duration of paralysis similar to that seen in the *ncs-1* null worms with an increase in time to paralysis and a decrease in duration. For *pifk-1* mutants, the phenotype was qualitatively similar to *ncs-1* null worms. The quantitative effect on time to paralysis was, however, much larger for the *pifk-1* mutant (increased by 296%) than what was seen with *ncs-1* null worms (increased by 74%). In the case of the two *arf-1.2* mutant lines tested, these did not give a consistently similar phenotype compared to that for the *ncs-1* null worms. For example, the *arf-1.2* (*ok1322*) strain had a time to paralysis that was not significantly different from N2 worms, while the other mutant strain (*ok796*) had a significantly reduced time to paralysis. The two *grk-2* mutant strains did not replicate the *ncs-1* null worm phenotype, but showed a decreased time to paralysis and no effect on its duration. From this analysis, we concluded that of those tested there are four potential target proteins for NCS-1 (*arf-1.1*, *pifk-1*, *trp-1* and *trp-2*) that could function in the pathway controlling the temperature-dependent behaviour based on similar mutant phenotypes in the onset and duration of paralysis in the TDL assay. Of these mutant strains, three (*arf-1.1*, *trp-1* and *trp-2*) had the most similar phenotype to the *ncs-1* null worms in the assay.

### Effect of double mutations on temperature-dependent locomotion

In order to investigate further the potential downstream targets in the TDL behaviour and to follow up the findings from single mutant strains, we generated double mutants to assess any genetic interaction. Genetic crosses were confirmed and validated through use of Polymerase Chain Reaction (PCR) analysis of genomic DNA. After confirming the presence of the *ncs-1* mutation in the *trp-1, trp-2* or *arf-1.1* mutant background, we analysed the behavioural phenotypes of the resulting double mutant lines in the revised TDL assay. Where multiple independent lines were generated, the data from their analysis were pooled together.

**Figure 4:**
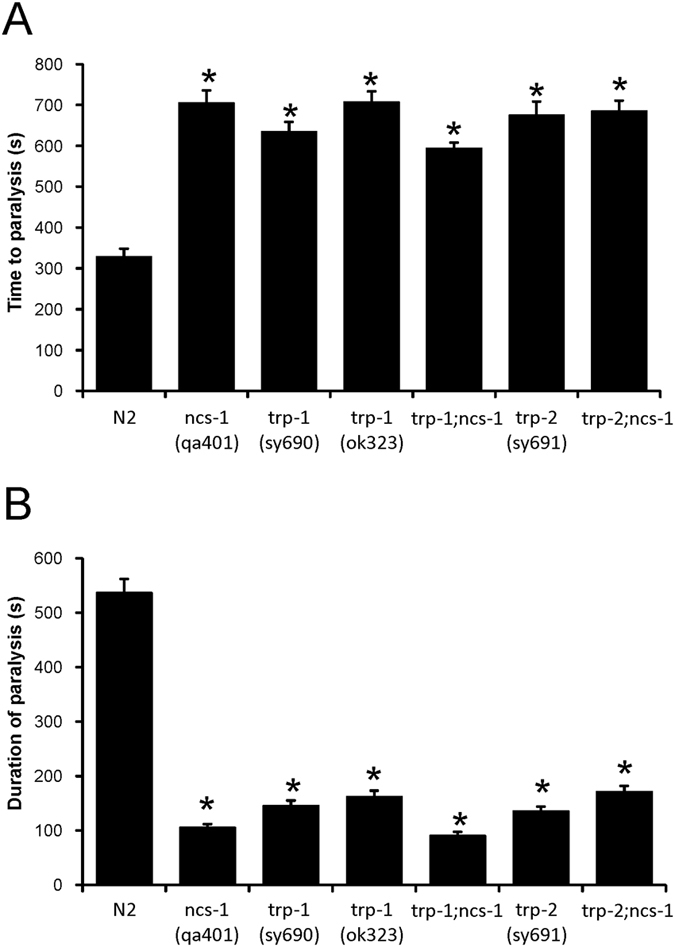
Assay of the temperature-dependent locomotion of the *ncs-1* mutation in the presence or absence of *trp-1* and *trp-2*. Temperature-dependent locomotion assays were carried out on various indicated strains of *C. elegans* to determine the onset and duration of paralysis after the shift to 28.5 °C. The double mutants were derived from genetic crosses of *qa401* with either *sy691* or *ok323*. Multiple animals (N ≥ 40) were tested for each strain and mean values for time to paralysis (**A**) and the duration of paralysis (**B**) were determined. All data are expressed as mean ± S.E.M. Statistical differences were identified by comparing averaged data to that of N2 wild-type worms, using one-way ANOVA with Dunnett’s correction for multiple comparisons (*p < 0.001 versus N2).

**Figure 5:**
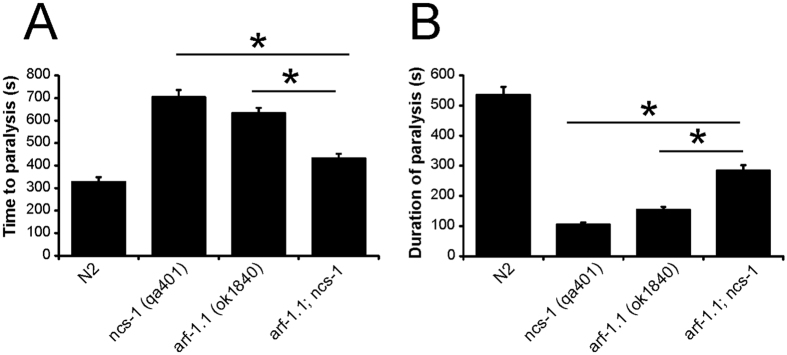
Assay of the temperature dependent locomotion of *arf-1.1*; *ncs-1* mutants. Temperature-dependent locomotion assays were carried out on various indicated strains of *C. elegans* to determine the onset and duration of paralysis after the shift to 28.5 °C. Multiple animals (N ≥ 60) were tested for each strain and mean values for time to paralysis (**A**) and the duration of paralysis (**B**) determined. All data are expressed as mean ± S.E.M. Statistical differences were identified by comparing averaged data to that of N2 wild-type worms, using one-way ANOVA with Dunnett’s correction for multiple comparisons (*p < 0.001).

The *trp-1; ncs-1* and *trp-2; ncs-1* double mutant strains showed defects comparable to N2 worms in their temperature-dependent locomotion phenotypes for both time to paralysis and duration of paralysis. Both phenotypic aspects were of essentially the same magnitude as that observed with single *ncs-1, trp-1* or *trp-2* single mutant strains (Fig. 4). The lack of any additive effect in the double mutants would be consistent with the proteins acting together or at least within a common pathway. The *arf-1.1; ncs-1* double mutant strains gave a different outcome in the revised TDL assay. The double mutants showed a significant increase in the time to paralysis (32% increase) and a decrease in the duration of paralysis (47% decrease) compared to N2 worms, but they also consistently (seen with four independently derived lines) showed an intermediate phenotype between those of the single mutants or N2 worms in the time of onset and duration of paralysis (Fig. 5). This intermediate phenotype seen with the double mutant is indicative of reciprocal sign epistasis, supporting the notion that NCS-1 and ARF-1.1 function together in the regulation of the revised TDL behaviour.

### Effect of inhibition of PIFK-1 on temperature-dependent locomotion

**Figure 6:**
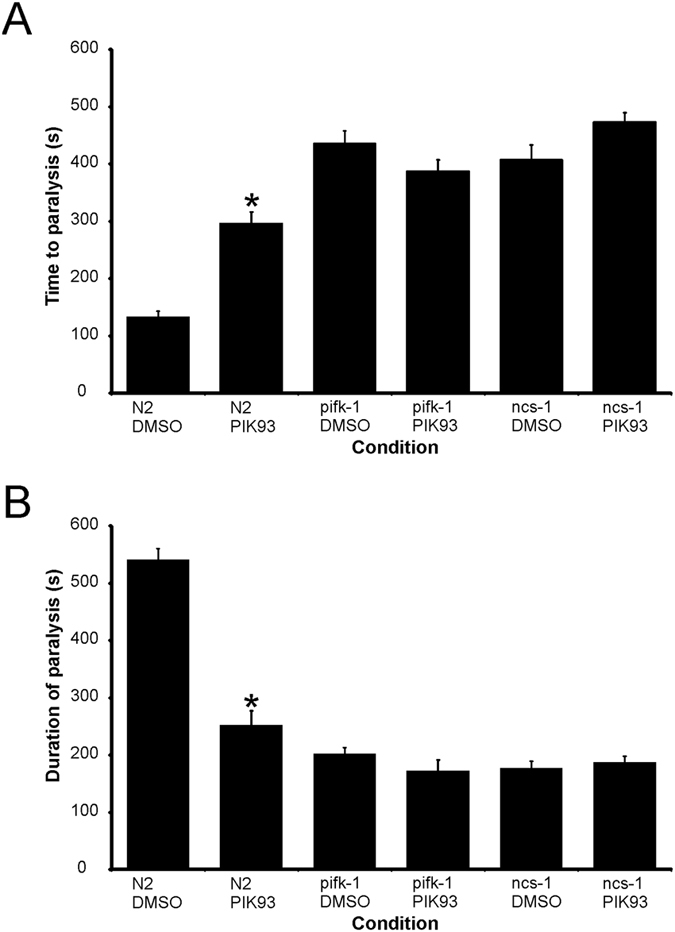
Effect of PIK-93 on temperature-dependent locomotion in N2, *pifk-1* and *ncs-1* null strains. Various worm stains as indicated were pre-treated for one hour with vehicle (1% DMSO) or 19 μM PIK-93. Temperature-dependent locomotion assays were carried out to determine the onset and duration of paralysis after the shift to 28.5 °C. Multiple animals (N = 30) were tested for each strain and mean values for time to paralysis (**A**) and the duration of paralysis (**B**) were determined. All data are expressed as mean ± S.E.M. Statistical differences were identified by comparing averaged data to that of N2 wild-type worms, using one-way ANOVA with Dunnett’s correction for multiple comparisons (*p < 0.001 versus vehicle control).

Since *ncs-1* and *pifk-1* reside close to each other on the X chromosome, generation of *ncs-1 pifk-1* double mutants would be problematical. We were able, however, to take advantage of an inhibitor of mammalian (PI4K) IIIβ inhibitor PIK-93^46^. Pre-treatment of N2 worms for one hour with 19 μM PIK-93 resulted in a longer time to paralysis and shorter duration of paralysis as seen with the *ncs-1* null worms (Fig. 6). To check the specificity of the action of the drug, we examined whether it would have any effect on *pifk-1* mutant worms. No difference in the behaviour of the *pifk-1* mutant worms was observed in drug-treated versus untreated worms (Fig. 6).

NCS-1 is able to activate mammalian (PI4K)IIIβ. Interestingly, PIK-93 also had no additional effect on the time to paralysis and shorter duration of paralysis in *ncs-1* null worms, supporting the involvement of *pifk-1* in the action of *ncs-1*.

### Effect of ARF1.1 rescue on temperature-dependent locomotion

**Figure 7:**
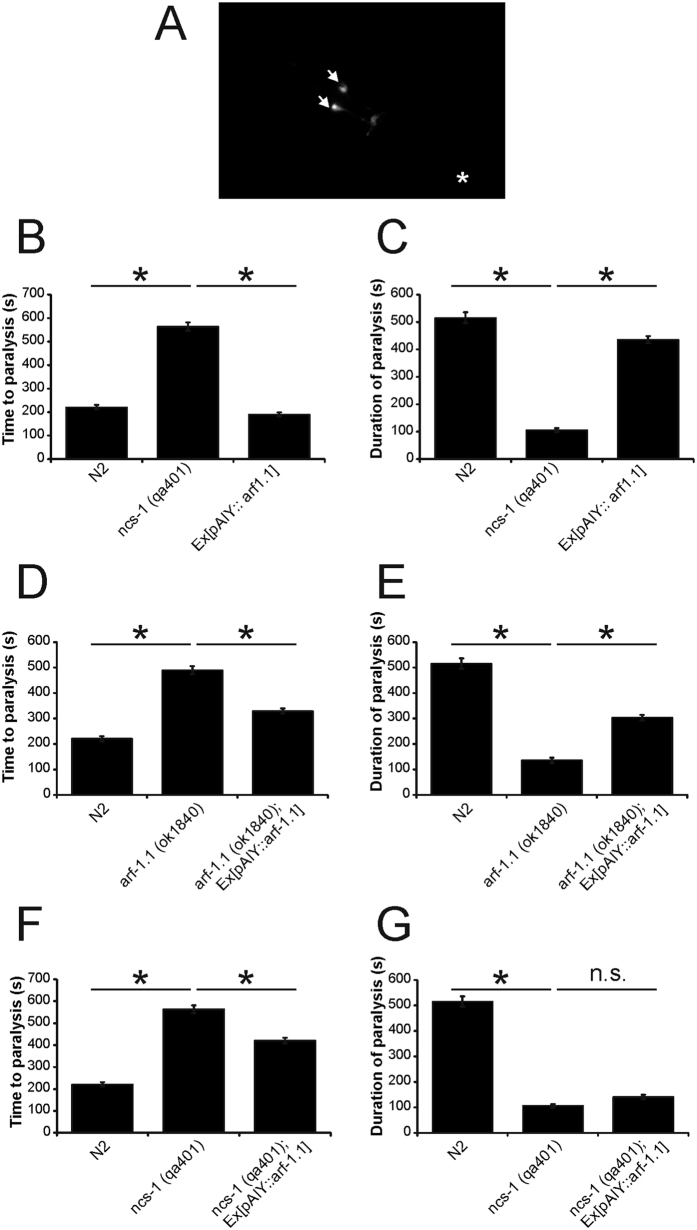
Assay of the temperature-dependent locomotion after ARF-1.1 overexpression or rescue. GFP expression is driven by *pAIY* specifically in the two AIY neurons indicated by arrows. No expression was observed elsewhere (**A**). The asterisk shows the position of the posterior tip of the imaged worm. Transgenic worms were generated to express ARF-1.1 within AIY neurons in a wild-type background (N ≥ 56) (**B**,**C**), in *arf-1.1* mutant worms (N ≥ 40) (**D**,**E**) or in *ncs-1* null mutant worms (N ≥ 56) (**F**,**G**). Temperature-dependent locomotion assays were carried out on various indicated strains of *C. elegans* to determine the time to the start of paralysis after the shift to 28.5 °C and the duration of paralysis. Multiple animals were tested for each strain and mean values for time to paralysis (**B**,**D**,**F**) and the duration of paralysis (**C**,**E**,**G**) were determined. Each transgenic data set presented used a conglomerate of at least 3 separate transgenic lines, which were pooled together. All data are expressed as mean ± S.E.M. Statistical differences were identified by comparing averaged data to those of N2 wild-type worms using one-way ANOVA with Dunnett’s correction for multiple comparisons (*p < 0.001).

The results from analysis of single and double mutants suggest that ARF-1.1 has a regulatory role in the control of the temperature-dependent behaviour that we have shown involves NCS-1 and that the two proteins may interact in the same signaling pathway. NCS-1 expression in the AIY neurons can rescue the phenotype in *ncs-1* null worms. In order to substantiate further the possible role of ARF-1.1, we aimed to examine whether expression of ARF-1.1 specifically in AIY neurons (Fig. 7A) was sufficient to rescue the *arf-1.1* mutant phenotype in the TDL assay. We first examined whether overexpression of ARF-1.1 in AIY neurons would have any effect by itself. Such over-expression had no major effect in the TDL assay (Fig. 7B,C): there was no effect on the time to paralysis, although there was a significant albeit small reduction in the duration of paralysis (15% reduction compared to a 79% reduction in *ncs-1* null worms). Expression of ARF-1.1 in AIY neurons of the *arf-1.1* mutant (Fig. 7D,E) resulted in a partial rescue of both the increase in time to paralysis and its duration. A similar extent of rescue was observed with pan-neuronal expression of ARF-1.1 driven by the *rab-3* promoter (Supplementary Fig. 2). These finding are consistent with the neuronal-dependent part of the phenotype of the *arf-1.1* mutant worms being attributed wholly to ARF-1.1 function in AIY neurons.

As we had noted a potential genetic interaction between *ncs-1* and *arf-1.1* from the analysis of the *arf-1.1; ncs-1* double mutant, we also examined the effect of overexpression of *arf-1.1* in AIY neurons in *ncs-1* null worms. ARF-1.1 overexpression in AIY (Fig. 7F,G) had little effect on the duration of paralysis, but significantly suppressed the delayed onset of paralysis in *ncs-1* null worms (90% increase versus the 151% increase in *ncs-1* null single mutants). This is again consistent with a functional interaction of the two proteins in the AIY neurons.

## Discussion

A key aspect to fully understanding how Ca^2+^ signaling is related to changes in neuronal function that lead to alterations in behaviour is the need to identify the components of the Ca^2+^ signaling pathways, including the essential proteins involved. Previous work has shown a requirement for the Ca^2+^ sensor protein NCS-1 in thermotaxis responses^17^ and in changes in locomotion rate following temperature elevation^19^. The ability to manipulate genetically protein expression in the worm in a specific manner, along with knowledge of the precise neurons involved in particular behavioural responses, provides an opportunity to investigate components of Ca^2+^ signaling pathways that function at the level of the organism.

*C. elegans* has long been used as a model organism to study cellular/genetic mechanisms that underlie neurosensory perception and neuroethology in general. The temperature-dependent locomotion (TDL) behaviour examined in this paper is particularly novel, in that it potentially reflects a cellular/genetic mechanism that allows the animal to adapt to harsh temperatures. The TDL phenotype is not only a thermosensory-driven behaviour, but it demonstrates adaptive responses within the context of a prolonged lethal environmental situation (high temperature). The nematode first continues to behave as normal during the initial exposure to the high temperature. In response to continued high temperatures, the animal stops moving – possibly to conserve energy or outlast the high temperature. As the high temperature continues, the animal then begins to move again, presumably with the revised goal of escaping the environment.

We have now developed our original temperature-dependent locomotion (TDL) assay from analysis of locomotion at a single time point at an elevated temperature^44^ to a quantitative assay that allows assessment of two key parameters by following the behaviour of individual worms. This gives information on the mean time to paralysis and the mean duration of the reversible paralysis occurring at the elevated temperature. The aim behind this was to provide the ability for a more discriminating comparison of temperature-dependent phenotypes between worm strains. The behaviour that we assayed uses known neural elements of the well-characterised thermotaxis response of worms^47^,^48^, including the involvement of the AFD and AIY classes of neurons^19^,^44^. The TDL behaviour differs, however, in its genetic requirement compared to those for both thermotaxis and temperature avoidance behaviour. The latter, for example, does not require NCS-1 function^17^,^49^ and thermotaxis is affected by genes not required for the TDL response^44^,^50^.

Multiple potential targets for NCS-1 regulation in response to Ca^2+^ signaling have been identified. The best characterised target in mammals is the dopamine D2 receptor, whose regulation by NCS-1^38^ has clear physiological roles in affecting behaviour in mice^26^,^51^. In most cases, however, the physiological significance of the potential identified targets is unclear. To date, no NCS-1 target protein interactions have been directly examined using biochemical or cellular assays for the worm proteins. The majority of the available information comes from study of mammalian proteins. The advantage of using *C. elegans* in the present study comes from the ability to use genetic approaches to study the involvement of the proposed target proteins in a behavioural phenotype at an organismal level, thus allowing physiological validation of the nature of the NCS-1 signaling pathway. Importantly, NCS-1 is highly conserved and the worm protein is 76% identical to the human protein. Clearly identifiable orthologues of potential target proteins are identifiable in worms with varying levels of sequence identity to the human orthologues of the proteins (ARF1.1 61% identity; ARF-1.2, 94% identity; GRK-2, 67% identity; PIFK-1, 31% identity; TRP-1 40% identity; TRP-2 47% identity), suggesting that these interactions would be likely conserved from worms to man, as known for many other examples of conserved protein-protein interactions. Note that the functional interaction of NCS-1 and PI4KIIIβ orthologues is conserved from yeast to mammalian proteins^16^,^30^. A three-way interaction between NCS-1, ARF-1 and PI4KIIIβ has been characterised using biochemical approaches and shown to affect secretory function in non-neuronal mammalian cells^30^. The significance of the NCS-1 interaction with ARF-1 for neuronal function *in vivo* has not been established. We set out therefore to test its relevance, along with the involvement of other potential NCS-1 targets in a defined behavioural assay, where NCS-1 function was known to be required in the pair of AIY neurons in the worm.

Analysis of single worm mutant strains showed that worms bearing mutation in four of the potential NCS-1 targets (*arf-1.1, pifk-1, trp-1* and *trp-2*) showed similar temperature-dependent behaviours to those of *ncs-1* null worms. One caveat, however, is that only single alleles were tested for *pifk-1*, and *trp-2*. We were able to show, however, that PIK-93, a specific inhibitor of PI4K IIIβ, resulted in a longer time to paralysis and shorter duration of paralysis, as seen with the *ncs-1* null worms and that this appeared to be a specific action on *pifk-1*. The lack of effect of PIK-93 on *ncs-1* null worms suggested an involvement of *pifk-1* in the action of *ncs-1* on temperature-dependent locomotion.

Based on the similar effects of mutations on time to paralysis and duration in the TDL assay, three genes (*arf-1.1, trp-1* and *trp-2*) had the most similar phenotype to the *ncs-1* null worms. This connection was further investigated by analysis of double mutant strains. The lack of additivity of the phenotypes of the *trp-1; ncs-1* and *trp-2; ncs-1* double mutant strains would be consistent with the proteins functioning in the same pathway. It was striking, however, that the *arf-1.1; ncs-1* double mutant showed indications of an intermediate phenotype compared to the single mutants. This type of genetic interaction is known as reciprocal sign epistasis, which is when two deleterious mutations or genes are more beneficial together than when they are alone, because the mutations impinge on the same process. These results, therefore, support the notion that the NCS-1 and ARF-1.1 function together in the regulation of the TDL behaviour.

It was important to establish that ARF-1.1 does indeed act in the same AIY neurons in which NCS-1 expression is required for normal TDL behaviour. ARF-1.1 is expressed in many cell types in the worm, but expression of ARF-1.1 in only the AIY neurons was sufficient to rescue the TDL phenotype, as effectively as pan-neuronal ARF-1.1 expression. This suggests that the neuronal-dependent part of the *arf-1.1* phenotype is entirely due to the ARF-1.1 function in the AIY neurons. Interestingly, overexpression of ARF-1.1 in AIY neurons in the *ncs-1* null worms was able to rescue the time to paralysis, but not the duration of paralysis. This latter result parallels the finding that AIY-specific expression of *ncs-1* is more effective in rescuing the “onset of paralysis” phenotype versus the “duration of paralysis” phenotype of the *ncs-1* mutants. Thus, this suggests that AIY neurons are more important in ARF-1.1- and NCS-1-dependent regulation of the onset of paralysis than in the regulation of its duration in the TDL assay.

This is the first description of this TDL assay (of onset and duration of paralysis) and the genes/cells that are involved. Both parameters of onset and duration of paralysis will presumably involve many steps, including temperature sensation, sensory information processing, motor output, as well as muscle contraction. Therefore, it is not surprising that each parameter could involve multiple genes and cells, some common and some distinct. The two parameters obviously have some common features, as NCS-1 expression in the AIY neurons alone is required for both phenotypes (Fig. 2). Some of our other data, however, point to other cells and genes being involved. Indeed, particular aspects of the locomotor circuitry could be important. Additionally, the *arf-1.1* rescue experiments indicate that driving expression in AIY alone only partially rescues the *arf-1.1* phenotype, but is as good at rescue as driving *arf-1.1* expression throughout the nervous system. This could point to an involvement of non-neuronal *arf-1.1* in the phenotype. Considerably more work would be required to investigate all aspects of the cells and genes involved.

Overall, the results presented here suggest an involvement of TRP-1, TRP-2, ARF-1.1 and PIFK-1 in the effects of increased temperature on locomotion. In addition, our findings support the notion that ARF-1.1 interacts with NCS-1 in AIY neurons within the Ca^2+^ signaling pathway that determines the effect of elevated temperature on *C. elegans* locomotion. This establishes that ARF-1.1 is a physiologically relevant partner in NCS-1 function.

## Methods

### *C. elegans* strains and culture

The existing *C. elegans* strains used in this project were Bristol N2 (wild-type) or mutant strains obtained, unless otherwise indicated, from the *Caenorhabditis* Genetics Centre (CGC) (University of Minnesota, USA). The mutant strains were: *arf-1.1(ok1840), arf-1.2(ok1233), arf-1.2(ok796), grk-2(rt97), grk-2(gk268), ncs-1(qa401), pifk-1(tm2348* from the National Bioresource Project for the “Experimental Animal Nematode *C. elegans”*, Tokyo, Japan*), trp-1(ok323), trp-1(sy690), trp-2(sy691).* The *ncs-1* rescue strains were described previously^18^. *C. elegans* strains were grown and maintained on standard nematode growth media^52^,^53^ supplemented with the relevant salts (NGM). Strains were maintained following standard protocol as described previously^54^ and maintained at 20 °C using *Escherichia coli* (*E. coli*) OP50 strain as the food source.

### Double mutant generation

Double mutant strains were generated by mating two existing strains and isolating the homozygous double mutant progeny by PCR-based genotyping.

Validation of double mutants was confirmed over multiple generations by PCR using the following primers:

ncs-1 forward, CAGTTGAGCATCGTTATTCTG

ncs-1 wild type reverse, CAGTTGAGCATCGTTATTCTG

ncs-1 mutant reverse, CCGTATTTGAACGTTGCTAC

trp-1 forward, CAACAGTTGCTCACCTCTATC

trp-1 wild type reverse, CCTCCGCTACCAACATTGGTTC

trp-1 mutant reverse, CGAATTTGTTGTGGAGGCAG

trp-2 forward, CACTGATGACGTGGATCGCAAGG

trp-2 reverse, CTAAGGGTGAAATATGACGAG

arf-1.1 forward, CATCGCCAACCAAGGAAAG

arf-1.1 wild type reverse, CCACATCAGACCTTCGTAGAG

arf-1.1 mutant reverse, CGACAGAGATCACCAAACATTG

The double mutant strains generated in this study were: *trp-1; ncs-1* (AMG 145–148), *trp-2; ncs-1* (AMG 128), *arf-1.1; ncs-1* (AMG 149–152).

### Plasmids used for *C. elegans* injections

The GFP marker plasmid for pan-neuronal expression of GFP (pRAB100 [P_*rab3::GFP*_]) was obtained from the Nonet Laboratory (Washington, University of St. Louis, USA). The AIY neuron-specific expression plasmid (PAIY::MCS) was obtained from the Hobert Lab (Columbia University Medical Center, N.Y.). All plasmids for injection were generated as Gateway DEST vectors (Life Technologies) to produce tissue-specific expression vectors as described previously^55^.

### Microinjection

Larval stage 4 to Day 1 adult *C. elegans*, including N2, *qa401* (*ncs-1* null), and RB1535 (*arf-1.1* null) were injected using a micropipette needle into their germline cells within the dorsal gonad. Injection mixtures contained a final concentration of 100 ng/μl DNA. Of this 100 ng, 10 ng/μl was the plasmid of interest (*ncs-1* or *arf-1.1* expression plasmid), 40 ng/μl of GFP marker plasmid and 50 ng/μl of empty pBluescript plasmid, the final volume was made up to 100 μl using dH_2_O. Multiple, independently-derived separate lines for each plasmid injection were generated and their phenotypes assessed. The transgenic strains generated in this study were: *Ex[PAIY::arf-1.1]* injected into a wild-type background*, arf-1.1 (ok1840); Ex[PAIY::arf-1.1], arf-1.1 (ok1840); Ex[P*_*rab3*_*::arf-1.1], ncs-1 (qa401); Ex[PAIY::arf-1.1].*

### Thrashing assay

Basal locomotion of all *C. elegans* strains used in this study was assessed. Day 1 adult hermaphrodite animals were transferred into a 50 μl droplet of Dent’s solution (140 mM NaCl, 6 mM KCl, 1 mM CaCl_2_, 1 mM MgCl_2_, 5 mM HEPES, pH 7.4 supplemented with bovine serum albumin at 0.1 mg/ml). Individual worms were allowed to acclimatise to the Dent’s solution for 10 minutes before thrashing rate was quantified. Thrashing was defined as one sinusoidal movement of the animal. Thrashing was measured over a 1-minute period for each individual worm. All experiments were completed at room temperature (~22 °C), using worms cultivated at 20 °C. Animals were first placed onto an unseeded NGM plate to remove any excess OP50 *E. coli* prior to locomotion being tested. None of the strains showed any differences in basal locomotion (Supplementary Table 1) indicating that differences in locomotion following temperature elevation could not be attributed to a general locomotion defect.

### Temperature-Dependent Locomotion Assay

A 35 mm petri dish lid was placed onto a Peltier effect thermoelectric plate (TEtech) to which a 150 μl droplet of Dent’s solution was added. Temperature throughout the assay was monitored manually in real time with a thermocoupler placed directly in the solution. Approximately five 1-day to 2-day old worms were placed into the Dent’s solution at room temperature (~20 ± 0.5 °C) and left to acclimatise for 5 minutes. Then current to the Peltier device was increased by heating the Dent’s solution to 27 °C in 1–2 minutes, at which point the timer was started manually. The temperature was allowed to rise over 1 min to a final temperature of 28.5 ± 0.5 °C. Two different parameters were measured for this assay: firstly, the time to paralysis, defined as a complete cessation of motility, using thrashing as a measure of locomotion; secondly, the onset of resumption of thrashing, defined as the time of the first forward thrashing movement. This was assessed while the worms were still at the elevated temperature and defined as the first appearance of two complete thrashes in a row. Multiple worms were observed in the same microscope field. Times for onset and end of paralysis were noted for each individual worm within each assay. Duration of paralysis was calculated by subtracting the onset of paralysis time from the end of paralysis time.

## Additional Information

**How to cite this article**: Todd, P. A. C. *et al*. Interaction of ARF-1.1 and neuronal calcium sensor-1 in the control of the temperature-dependency of locomotion in *Caenorhabditis elegans*. *Sci. Rep.* **6**, 30023; doi: 10.1038/srep30023 (2016).

## Supplementary Material

Supplementary Information

Supplementary Video 1
