# Supplementary material for: Interaction of ARF-1.1 and neuronal calcium sensor-1 in the control of the temperature-dependency of locomotion in Caenorhabditis elegans
Source: Sci Rep. 2016 Jul 20;6:30023. doi: 10.1038/srep30023 (PMC4951722; doi:10.1038/srep30023)
Supplement: Supplementary Information [file srep30023-s1.pdf]

Interaction of ARF-1.1 and neuronal calcium sensor-1 in the control of the temperature-dependency of locomotion in *Caenorhabditis elegans*

Paul A.C. Todd, Hannah V. McCue, Lee P. Haynes, Jeff W. Barclay and Robert D. Burgoyne

Supplementary data

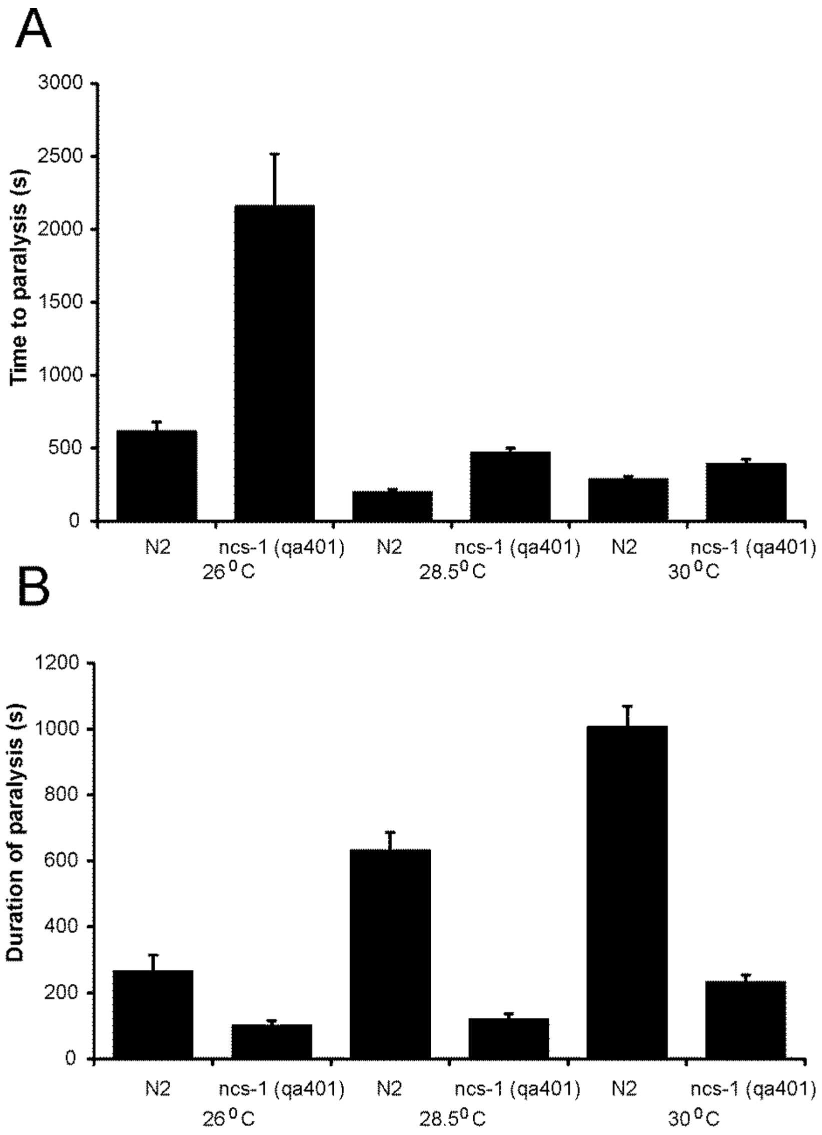

Supplementary Figure 1: Preliminary analysis of the temperature-dependent locomotion of N2 and null *ncs-1* worms.

The locomotion of worms was initially assessed at 20°C and then followed after a temperature shift to 26°C, 28.5°C or 30°C. Multiple animals were tested for each strain and mean values for time to paralysis (A) and the duration of paralysis (B) were determined. Note that all N2 worms became paralysed at all three elevated temperatures, but at 26°C only 33% of *ncs-1* null worms became paralysed. The numbers of animals used for each strain were ≥ 15.

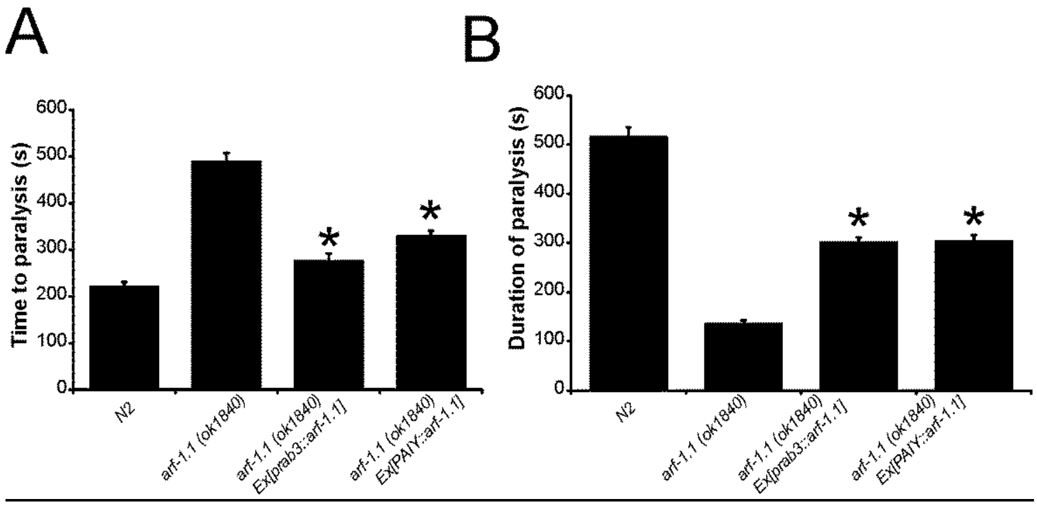

Supplementary Figure 2: Analysis of the temperature-dependent locomotion of *arf-1.1* mutant and *arf-1.1* rescue strains.

Transgenic worms *arf-1.1* mutant were generated to express ARF-1.1 pan-neuronally under the control of the *rab-3* promoter or only within AIY neurons. Temperature-dependent locomotion assays were carried out on various indicated strains of *C. elegans* to determine the time to the start of paralysis after the shift to 28.5^{o}C and the duration of paralysis. Multiple animals (N ≥ 40) were tested for each strain and mean values for time to paralysis (A) and the duration of paralysis (B) were determined. Each transgenic data set presented used a conglomerate of at least 3 separate transgenic lines, which were pooled together. All data are expressed as mean ± S.E.M. Statistical differences were identified by comparing averaged data to those of N2 wild-type worms, using one-way ANOVA with Dunnett's correction for multiple comparisons (*, p<0.001 compared to arf-1.1(ok1840)).

Supplementary Table 1. Basal thrashing rates for worm strains used in this study.

Thrashing was defined as one sinusoidal movement of the animal and was measured over a 1 minute period for each individual worm. All experiments were completed at room temperature (~22°C), using worms cultivated at 20°C (n≥ 40).

Strain	Mean thrashes	SEM
N2	90.833	2.7961
ncs-1 (qa401)	98.267	2.4024
pifk-1 (tm2348)	109.267	1.4819
trp-1 (ok323)	104.450	1.2934
trp-1 (sy690)	101.650	1.0188
trp-2 (sy691)	96.275	1.0640
arf-1.1 (ok1840)	109.267	1.4819
arf-1.2 (ok1322)	92.425	0.9792
arf-1.2 (ok796)	92.100	1.0584
grk-2 (rt97)	94.150	0.9416
grk-2 (gk268)	94.100	1.0444
trp-1 & ncs-1 DM	97.456	0.6454
trp-2 & ncs-1 DM	96.450	1.9993
arf-1.1 &ncs-1 DM	94.724	1.7275
ncs-1 (qa401) Ex[pNCS-1::NCS-1]	102.750	0.7204
ncs-1 (qa401) Ex[pAIY::NCS-1]	102.450	0.7729
N2; Ex[pAIY::Arf-1.1]	96.317	0.4983
arf-1.1 (ok1840); Ex[pRab3::arf-1.1]	94.017	0.5868
arf-1.1 (ok1840); Ex[pAIY::arf-1.1]	96.733	0.7467
ncs-1 (qa401); Ex[pAIY::Arf-1.1]	97.017	0.6442

Supplementary Table 2

Key NCS-1 targets and their orthologues in C. elegans

Human protein	*C. elegans* Gene name	sequence	Notes
PI4KIIIb	***pifk-1***	**F35H12.4**	expressed in many neurons
Cav2.1 channel	*unc-2*	T02C5.5	expressed in neurons but not AIY
D2 receptor	*dop-2*	K09G1.4	expressed in neurons but not AIY
	dop-3	T14E8.3	expressed in neurons but not AIY
ARF1	***arf1.1***	**F45E4.1**	expressed in many neurons
	arf-1.2	**B0336.2**	expressed in many neurons
IP3 receptor	*itr-1*	F33D4.2	poor expression in neurons
IL1RAPL1	*no orthologue*		
TRPC1/TRPC5	*trp-1*	ZC21.2	expressed in neurons but not AIY
	trp-2	**R06B10.4**	expressed in many neurons
GRK2	***grk-2***	**W02B3.2**	expressed in many neurons

Information on genes and expression is derived from Wormbase (www.wormbase.org)

Supplementary Video 1

A video of a group of worms during the temperature-dependent locomotion assay showing the complete paralysis of some but not all worms at the elevated temperature and the later recovery of locomotion by the paralysed worms.
